# Microbial Evolution in Allodapine Bees: Perspectives From Trophallactic, Socially Plastic Pollinators

**DOI:** 10.1111/eva.70137

**Published:** 2025-07-27

**Authors:** Simon M. Tierney, Thomas C. Jeffries, Hauke Koch

**Affiliations:** ^1^ School of Science Western Sydney University Richmond New South Wales Australia; ^2^ CSIRO Environment Canberra Australian Capital Territory Australia

**Keywords:** microbial transmission, network microbiome, network trophallaxis, pathogen susceptibility, pollination services, social immunity

## Abstract

This review seeks a deeper functional understanding of wild bee microbiomes by focusing on a tribe of bees where natural history and behavioral ecology are well known but investigations of microbiology are just beginning. Opportunities to improve our future knowledge of pathogens to insect pollinators are explored—which have broad ramifications for crop pollination services, considering the current overdependence on a few managed species that face a multitude of health threats. The bee tribe Allodapini (Apidae: Xylocopinae) has the potential to offer comparative insights on the evolution of bee microbiomes, owing to a unique combination of life history traits relevant to pollination service delivery across sub‐Saharan Africa, Southern Asia, and Australia. Allodapines exhibit facultatively social colony organization that offer evolutionary perspectives on the formation of group living not afforded by obligately eusocial insects, which have already transgressed the solitary‐social threshold. Progressive provisioning of brood (in the absence of brood cells) facilitates a network exchange of nutrients (via trophallaxis) that we speculate may culminate in an intra‐colony “network microbiome”. A literature review of pathogenic (bacterial, fungal, viral, and protozoan) associates of allodapine bees reveals considerably less research than for carpenter (*Ceratina*, *Xylocopa*), bumble (*Bombus*), and honey (*Apis*) bees. Interrogation of published genomes (*Exoneura*, *Exoneurella*) discovered novel microsporidian and protozoan parasites and relatives of known bee bacteria (*Commensalibacter*, *Sodalis*). Some *Xylocopa* exhibit microbial profiles typical of corbiculate bee core gut microbiomes, but no comparative evidence among allodapines was found. Allodapines visit flowers of 13 horticultural crops (fruits, vegetables, oilseeds, tree‐nuts) and 50 native genera (predominantly Myrtaceae, Proteacae, Myoporaceae, Goodeniaceae). The ability to parse intrinsic and extrinsic factors influencing microbiome patterns within and between species means that allodapine bees provide the opportunity for an integrated approach to bee socio‐eco‐evo‐immunology.

## 1 Introduction

Bee microbiomes are relatively simple assemblages that enable organismal health to be correlated with behavior with few artifacts and high confidence. Hence, there is wide scope to assess microbial–host‐behavioral relationships in social bees that exhibit caste‐specific behavior. While highly organized colonies of corbiculate bees come to mind, a variety of other facultatively social, casteless, and subsocial lineages exist (Michener 1974; Dew et al. 2016) and will be considered in this review.

A holistic functional understanding of bee microbiomes across the bee phylogeny (~20,000 species) can be obtained by the collection of independent empirical data on pathogens, environmental sources, and host‐behavioral transmission. Such comparative insights have important applied outcomes for pollination services to food crops—especially given: (i) largely undocumented free pollination services provided by unmanaged (wild) native bees; and (ii) heavy reliance on Western honey bees that are increasingly under threat from a variety of pathogenic microorganisms: viruses, bacteria, fungi, and protists. For example, native allodapine bees are the second most commonly reported pollinators of apple in Australasia (Tierney et al. 2023), yet we know relatively little about their microbial associates. Therefore, an improved understanding of the evolutionary history of the microbiota that influence bee health has practical implications for agricultural industries and human food security.

In this review, we focus on the microbiota of allodapine bees (Apidae: Xylocopinae: Allodapini) from comparative evolutionary and ecological perspectives. Allodapines are common and widely distributed pollinators that exhibit facultative sociality—the ability to switch between single‐female or social nest assemblages (Schwarz and Tierney 2020) – in addition to a combination of unusual traits: progressive provisioning of brood in undivided linear stem nests with no brood cells and reciprocal feeding to larvae and other adults by regurgitation (“trophallaxis” per Wheeler 1918). In this sense, allodapine nesting biology may be more similar to stem nesting ants (e.g., *Pheidole*, *Pseudomyrmex*) than other bee lineages.

Direct exchange of nutrients under group living conditions has major implications for the evolution of microbial assemblages associated with allodapine bees—factors that have clear impacts on health (immunity and pathogen susceptibility) at colonial, population, species, and community scales. More broadly, transmission of nutrients within a nest via trophallactic mutual feeding has contributed to concepts of a “social stomach” (Sleigh 2002) as a promoter of the evolution of eusocial colony organization in insects. The advancement of “multi‐omic” approaches to bee health: assessing interactions between a bee's gut, brain, and behavior (Zhang et al. 2022) can help us address research questions (Box 1) that integrate an organism's behavioral ecology with its microbial health—which we explore below.

BOX 1 **Questions and future directions.**

**Table:** 

Outstanding questions	Research directions
Network microbiome	
Do allodapine bees exhibit network microbiomes?	Ontogenetic catalogue of microbiomes & intracolonial transmission experiments
Core microbiomes	
Why do some socially polymorphic bees harbour corbiculate core‐microbiomes?	Environmentally controlled comparative assessments
Cobiont impact	
What are the impacts of bacterial & eucaryotic parasites on bee health?	Ethologically‐based screens of microbiomes & by‐catch from whole genomes
Social immunity	
How does the presence of cobionts potentially link to social evolution?	Phylogenetic tests that isolate factors: social organization, group‐size, nest environment
Evolutionary ecology	
Does foraging behavioral ecology influence selection of the microbial physiochemical niche?	Move from descriptive species‐level patterns to advanced functional understanding of socio‐eco‐evo‐immunology
Evolutionary applications	
How can native bee microbiomes inform interventions for managed bee populations?	Understanding microbiome function to counter impacts of anthropogenic stressors

### 1.1 Scope of the Review

We begin by outlining the agroecological impact of allodapine bees and how their unusual behavioral ecology (Table 1) is relevant to understanding microbial ecology, immunity and pathogen susceptibility. Hence, a deeper explanation of the functional traits (nesting biology, social organization, nutrient transmission) that are likely to influence microbiota and their transmission is provided. A ubiquitous ‘network’ of nutrient transmission (network trophallaxis) among adults and larvae has previously been noted (see Section 3.3), and a key aim of this review and future research is to explore whether this translates into a network‐microbiome within allodapine colonies. We then undertake a systematic review of allodapine bee microbial cobionts within published literature and genomic data to improve our understanding of allodapine immunity and pathogen susceptibility. The review does not canvas intracellular bacterial parasites, such as *Wolbachia*. Discussions are framed with comparative reference to tribes within the subfamily Xylocopinae and other facultatively social bee lineages. In conclusion, we highlight the importance of developing this knowledge base and areas of greatest research potential (Box 1).

**TABLE 1:** Functional traits.

		Nesting biology			Social organization	Microbial transfer	
		Architecture	Founding	Feeding		Oral trophallaxis	Gut
Apidae							
Xylocopinae							
Allodapini Nonparasitic genera	Allodapine bees	Wooden stems No brood cells	Single Cofounded	Progressive	Facultative: Eusocial Semisocial Casteless Subsocial	Networked: Between all adults All adults to all larvae	Unknown (fecal removal)
Ceratinini *Ceratina*	Small carpenter bees	Wooden stems Brood cell partitions	Solitary	Mass	Facultative: Semisocial Solitary	Directional: Forager to nest guard Mother to callow daughter	Unknown (fecal removal)
Xylocopini *Xylocopa*	Large carpenter bees	Wooden stem nest Brood cell partitions	Solitary	Mass	Facultative: Semisocial Solitary	Directional: Forager to nest guard Mother to callow daughter	Unknown
Apinae							
Apini *Apis*	Honey bees	Cavity Brood cell combs	Fission	Progressive	Obligate: Eusocial	Multidirectional: Worker to worker Queen to worker Worker to drone	Vertical trans. Comb contact Bee bread
Bombini *Bombus*	Bumble bees	Cavity Dynamic cells	Solitary	Progressive	Obligate: Eusocial Subsocial	Absent	Vertical trans. Social contact Fecal contact

*Note:* Life history and behavioral traits influencing microbiota of allodapine, ceratinine, xylocopine, apine and bombine bees within the family Apidae. Feeding refers to brood provisioning behavior. Transfer of gut microbiota is unknown for bees from the subfamily Xylocopinae although allodapine and ceratinine bees actively remove larval fecal pellets.

## 2 Agroecological Implications

Motivation to extend the breadth of bee microbial research (well beyond *Apis* and *Bombus*) stems from historical declines in honey bee populations due to parasites and associated viral pathogens (Chapman et al. 2023). This is critical given the dependence of human food security on very few bee species for agroecological pollination services—the result of cultivating global food crops well beyond their natural distributions. Subsequently, there is likely to be a co‐evolutionary mismatch with respect to native bee pollinators in biogeographically discrete regions (Brown and Cunningham 2019; Tierney et al. 2023). For example, cultivated apple is a Central Asian plant and out of the four most common bee genera that visit Palaearctic apple orchards (*Apis*, *Bombus*, *Osmia*, *Andrena*), none naturally occur in Australasia, where allodapine (*Exoneura*) and halictine (*Lasioglossum*, *Homalictus*) bees are the most common native pollinators (Tierney et al. 2023).

Allodapine bees are known to visit various horticultural crop groups (Table S1), including fruits (berries, pome, tropical), vegetables (fruiting, leafy, root and tuber, stalk and stem), oilseeds, and tree nuts—*per* Codex classifications (Food and Agriculture Organization 1993). Most research has occurred in India and Australia (Table S1), tropical cosmopolitan (*Braunsapis*) and temperate Australian (*Exoneura*, *Brevineura*) bee genera feature in these reports, despite the fact that Allodapini originated from and are most diverse in the Afrotropics (Michener 2007; Tierney et al. 2008; Schwarz and Tierney 2020). While relatively less applied pollination research has been undertaken in African countries (see Eardley et al. 2009; Tierney et al. 2023), this region perhaps holds great potential to provide future insight on pollination services given relative generic diversity (excluding parasites): India, 1 genus; Australia, 4 genera; Africa, 8 genera (Chenoweth et al. 2008; Tierney et al. 2008).

From a functional perspective, allodapines appear to be very effective pollinators of food crops. For apples grown in Australia, *Exoneura* deposited the most apple pollen during single visits to flowers (*D* = 231.25), followed by *Lasioglossum* (*D* = 161.89); both of which exceeded stingless (*D* = 57.07) and honey bees (*D* = 99.92) (Bernauer, Tierney, et al. 2022; Tierney et al. 2023). Hence, there is considerable merit in exploring the microbiology and health of such alternate pollinators, given the tendency to visit a range of crop flowers (Table S1) and broad biogeographic distribution.

## 3 Allodapine Bee Traits Influencing Microbiota

**FIGURE 1:**
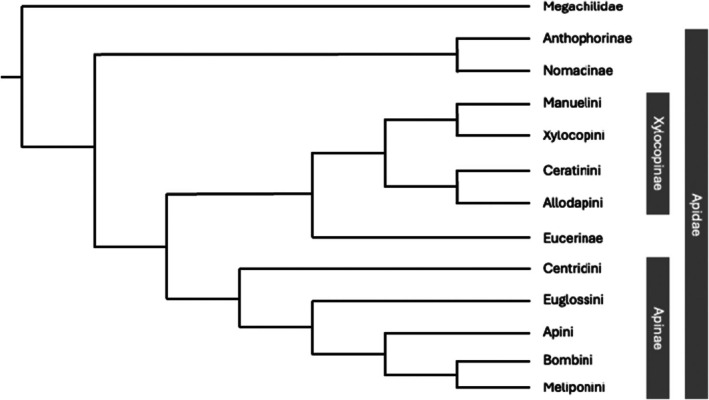
Higher level cladogram of the Apidae. Indicates phylogenetic relationships between the tribes of Xylocopinae and Apinae. Branching patterns and classifications *per* Bossert et al. (2019), to the exclusion of Ctenoplectrini and Tetrapidini. All presented nodes are maximally supported (posterior probability) under Bayesian inference of a concatenated 80% matrix of combined genomic and transcriptomic loci.

Tribe Allodapini is related to the small and large carpenter bee tribes Ceratinini, Xylocopini and the relictual Manueliini, which have historically comprised the Xylocopinae (Figure 1) (Michener 2007); but see Bossert et al. (2019) for the hypothetical inclusion of Ctenoplectrini and Tetrapediini. Allodapines are broadly distributed throughout the Afrotropical, Indomalayan, and Australasian realms and have dispersed across considerable oceanic barriers (Fuller et al. 2005). Allodapini exhibits greater generic‐level diversity (~14 genera, ~160 species) compared to the remaining tribes of Xylocopinae (monogeneric) and greater diversity in larval morphology than all other bees combined (Michener 1977). A summary of atypical life history traits relevant to microbiology is summarized in Table 1 and canvassed in the sections below.

### 3.1 Nesting Biology

**FIGURE 2:**
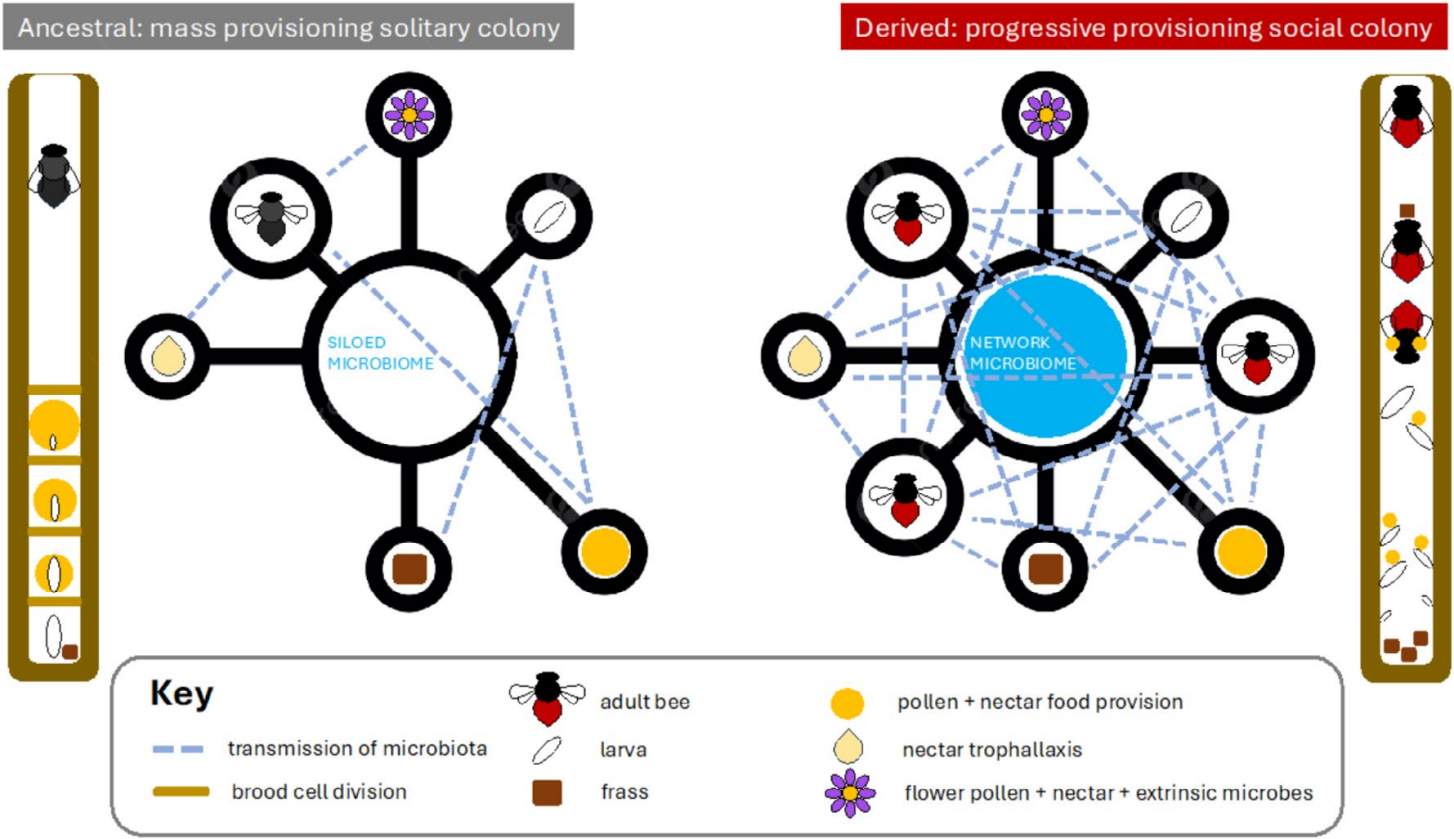
Microbial networks. Hypothesized microbial transmission pathways in stem nesting bees of the subfamily Xylocopinae, comparing an ancestral mass provisioning solitary bee colony with a derived progressive provisioning social bee colony. Ancestral lineages (Xylocopini) construct brood cells and have no contact with immature brood until they eclose as adults—leading to a relatively “siloed microbiome”. Derived lineages (Allodapini) lack brood cell divisions, are in frequent physical contact with immature brood, engage in network trophallaxis and adults eject larval frass from nests—these combined traits should contribute to a highly interconnected intracolonial “network microbiome”. Bold circles and bars represent the various components contributing to either a Siloed or a Network microbiome.

The etymology of subfamily Xylocopinae derives from Ancient Greek (Liddell et al. 1996): *xylokópos* (“hewing” wood); Allodapini from *allodapós* (“belonging to another land, foreign”); Ceratinini from *cĕrătĭna* and *keratinos* (“concerning horns” or “made of horn”). In all tribes, linear nests are excavated from plant stems, branches or trunks. Allodapini are further distinguished from other bees because immatures are reared in open nest chambers in the absence of brood cell divisions—a derived diagnostic trait (Figure 2). Hence, individual larvae are progressively provisioned with food as they develop. Larvae defecate during development, and larval faecal pellets are collected and removed by adult females (Michener 1971). Whereas, the ancestral means of mass‐provisioning brood in bees and wasps is to: (1) deposit enough nutrients for an immature to reach adulthood in a single brood cell; (2) lay an egg on top of the pollen/nectar mass‐provision; (3) seal the brood cell.

### 3.2 Social Organization

The lack of brood cells in allodapine nests means that any single adult female rearing immatures is sub‐social by definition, rather than “solitary” per se (Michener 1969, 1974, 2007). The addition of adults to such assemblages immediately renders the colony ‘social’ because their mere presence provides some degree of alloparental care in the form of brood protection, regardless of whether this behavior is directly or indirectly altruistic. The development of reproductive or worker‐like social castes is deemed facultative among allodapines because females are totipotent and never lose the ability to lay eggs should a reproductively dominant “queen” senesce. Species within this tribe vary from being predominantly subsocial, to semisocial and eusocial depending on the relatedness between queens and workers (sister–sister or mother–daughter assemblages). Alternatively, multifemale nests may be ‘casteless’ (Dew et al. 2016, 2018a, 2018b), where there is a distinct lack of reproductive skew and multiple egg layers engaging in alloparental care for the brood of other nestmates—the equivalent to communal nesting such as found among many mass provisioning halictine bees (Wcislo and Tierney 2009).

Because any female can physiologically reproduce, sociality is “facultative” as there are no constraints to independent nesting. The evolution of sterile worker castes represents a long‐standing conundrum of Darwinian evolutionary theory, for which facultative and incipiently social bee species provide unique insights (Michener 1974). Subsequently, both allodapine and halictine bees have provided comparative insights for the exploration of evolutionary signals that promote cooperative behavior among animals (Schwarz et al. 2007). Whereas, social insects with very distinct and highly specialized castes (e.g., ants, corbiculate bees) have long passed the threshold where meaningful evolutionary signal can be discerned with regard to the factors that may have promoted the origins of social group living. The facultative nature of allodapine social colonies means they can easily be manipulated (e.g., Bull et al. 1998) or observed along environmental gradients (e.g., latitude, altitude—Cronin and Schwarz 1999, 2001; Bernauer et al. 2021) where the climate may preclude eusocial colony formation by restricting the duration of brood rearing season and preventing an overlap of generations. Some allodapines continuously lay eggs throughout the brood rearing season (staggered rather than pulses of cohorts) resulting in frequent opportunities for eusociality to form (e.g., Tierney et al. 1997).

It is important to remember that this flexibility in allodapine social organization has persisted for extensive periods of geological time—with an inferred tribal origin of at least ~45–50 Mya (Tierney et al. 2008). Only one eusocial species with distinct morphological caste differentiation has arisen—“majors” and “minors” of *Exoneurella tridentata* among the Australian exoneurine lineage (Houston 1977). A handful of other species consistently exhibit marked size‐based reproductive differentiation (e.g., *Hasinamelissa* and *Brevineura*), which raises questions of: (1) why these facultative assemblages have persisted for so long; and (2) why only one species has become canalized into eusocial nesting with relatively large colonies containing up to ~40 adult females and ~80 immature brood; whereas all other species for which data exist never exceed 20 adult females or 40 brood per nest (Dew et al. 2012).

It was initially thought that the catalyst in allodapine social evolution was related to progressive brood provisioning behavior (Michener 1977, 1985, 1990). However, the discovery of social nesting in *Macrogalea* and molecular phylogenetic revisions of the tribe indicated that progressive provisioning is ancestral (Tierney et al. 2002, 2008); these findings altered the paradigm for how social evolution was perceived to have evolved within Allodapini. Ancestral state reconstructions exploring strategies of callow females (reproduce, forage or wait for opportunities to assume reproductive dominance), imply that the most recent common ancestor of species with discrete workers castes most likely “waited” for opportunities to become the reproductive dominate female (queen) within a social queue (Schwarz et al. 2011). This is very different to developmental groundplan models for the origin of worker castes that imply a disassociation of gene networks linked with foraging and egg laying behavior in solitary ancestors (West‐Eberhard 1996; Amdam et al. 2006). This fundamental difference highlights the fact that allodapine sociality has likely evolved on an independent evolutionary pathway from the majority of social insects. It is precisely these alternate behavioral modes that renders allodapine microbial ecology and evolution as topics worth deeper understanding.

### 3.3 Nutrient Transmission Between Individual Bees

The combination of nest architecture and progressive brood rearing behaviors likely accounts for the aforementioned larval diversity, including ventral appendages (tubercules, pseudopods), given that larvae can freely move within the nest and need to manipulate food directly transferred from adult foragers. This continual contact between feeding immatures and adults includes the combination of nectar with collected pollen to create a food provision that is directly provided to the venter of feeding‐stage larvae to consume (Michener 1971, 1972a).

One of the notable traits among eusocial bees from the subfamily Apinae (and ants) is the direct transfer of regurgitated nectar‐water among adults—termed “trophallaxis” (Michener 1974). Ethological studies of social allodapine bee intra‐colony behaviors report species of various genera (*Allodape*, *Braunsapis*, *Exoneura*) engaging in trophallaxis (Michener 1972a, 1972b; Mason 1988; Melna and Schwarz 1994). Mason (1988) described allodapine trophallaxis as a “network” with all individuals receiving and donating nutrients (see Figure 2). Trophallaxis often involves buccal contact, which may also be involved in communication between individuals given that all members of the colony made buccal contact, most frequently among guard bees (Melna and Schwarz 1994).

Trophallaxis is also reported in the sister tribes Ceratinini (Sakagami and Maeta 1984, 1987) and Xylocopini (Rau 1933; Anzenberger 1977; Gerling et al. 1983). Hence, the long lifespans of adults in combination with trophallactic nutrient exchange has long been proposed as a potential factor promoting social evolution among the Xylocopinae (Michener 1972b). It has also been noted that trophallaxis in *Ceratina* and *Xylocopa* predominantly occurs between a returning forager and a nest guard, whereas allodapine trophallactic exchange can happen anywhere within the nest (Mason 1988). This raises the question of whether allodapines are likely to exhibit what we hypothesize may constitute a “Network Microbiome” (Figure 2)—as distinct from the “Siloed Micriobiome” exhibited by solitary nesting ancestral lineages of Xylocopinae. An allodapine ‘network microbiome’ would represent an independent parallel to the conserved vertically transmitted core‐microbiomes in honey and bumble bee hives of the subfamily Apinae (Figure 1; see Section 4 ‘*Transmission of microbiota*’ below).

Outside of Apidae, the only reported instances of trophallaxis among bees occur in two genera of Halictidae: the casteless (communal) ground nesting *Lasioglossum hemichalceum* (formerly *L. erythrurum* : Kukuk and Crozier 1990; Kukuk 1992); and two species of facultatively eusocial stem nesting *Megalopta* (Wcislo and Gonzalez 2006). Among ancestral lineages (remembering that bees arose from wasps ‐ Branstetter et al. 2017) multifemale nests of the sphecid wasp *Microstigmus nigrophthalmus* also engage in trophallaxis and buccal contact among adult nestmates (de Melo and Campos 1993). This is noteworthy from a comparative evolutionary perspective, because *Microstigmus* is the only sphecid genus to exhibit eusocial colony organization with female body‐size differentiation, whereas all remaining sphecids form casteless groups or nest solitarily (Wcislo and Tierney 2009)—suggesting a repeated co‐occurrence of these two traits in a phylogenetically independent lineage.

While all ants are eusocial, only half of ant genera engage in trophallaxis – for which mouth‐to‐mouth transmission is speculated to be a correlate of ecological dominance (*reviewed by* Meurville and LeBoeuf 2021). Whereas, these recurring co‐occurring traits (eusociality + trophallaxis) among bees and wasps have historically prompted theoretical links between trophallaxis as a form of “extended parental care” facilitating the origin of eusociality. However, the role of trophallaxis in determining the microbiomes of “weakly” social bees was deemed equivocal, based on the limited available evidence (see Wcislo 2016). Although, the network‐trophallaxis exhibited by allodapine bees (Figure 2) had not been considered in such contexts. Casteless societies of *Lasioglossum hemichalceum* may provide the closest parallel to allodapines in that there appears to be no matrifilial or caste‐based hierarchical dominance structure to trophallactic transfer of nutrients between adults, albeit *Lasioglossum* are mass provisioning halictine bees with very different natural histories (*cf*. Allodapini).

## 4 Transmission of Microbiota

Microbes associated with bees comprise a wide variety of viruses, bacteria, fungi, and protists that can act in a commensal or pathogenic manner with regard to bee health (reviewed by Evans and Schwarz 2011; Engel et al. 2016). How the microbiota of the allodapine bees are transmitted is yet to be investigated and represents a motivation for this review and a focal point for future studies (Box 1). In addition to network trophallaxis among adult and larval colony members (detailed in Section 3.3) and the physical transportation of larvae within nests (Michener 1971), allodapines are also unusual in that larvae defecate in the open nest tunnel. The absence of brood cells renders nest tunnels a shared space, and larval fecal pellets are removed from the nest by adults well before pupation. Some halictine and ceratinine bees access brood cells to remove larval feces prior to adult eclosion (Michener 1974). However, African and Australian allodapine genera defecate while still feeding (Sakagami 1960), and this differs from most other bee lineages where larvae do not excrete feces until growth has completed. This quiver of peculiar brood maintenance behaviors could facilitate a multi‐directional transmission of gut microbiota, given the high degree of connectedness between individual colony members—providing further incentive to investigate the presence of network microbiomes in allodapine bees.

Research in other bee taxa regarding the link between microbiome transmission and sociality is summarized below for context. Early studies found that the eusocial corbiculate bee tribes of honey bees (Apini), bumble bees (Bombini) and stingless bees (Meliponini) harbored a consistent, specialized microbiome in the adult gut (Martinson et al. 2011; Kwong et al. 2017). Few “phylotypes” dominated the microbiome, including Proteobacteria (*Snodgrassella* sp. & *Gilliamella* sp.), Firmicutes (Firm‐4 = *Bombilactobacillus* sp. and Firm‐5 = *Lactobacillus* sp.), Actinobacteria (*Bombiscarvdovia*), and Bacteroidetes (*Apibacter* sp.). In contrast, the adult gut microbiomes of solitary bees show high variability and dominance of unspecific, environmentally acquired bacteria, and a lack of the “corbiculate core” (Martinson et al. 2011; McFrederick et al. 2012, 2013; Fernandez De Landa et al. 2023). This supported a view of sociality being a key factor for the evolution of specialized and potentially beneficial microbiomes in bees (Koch and Schmid‐Hempel 2011), likely by enabling consistent vertical transmission from parent to offspring generations within the shared nest environment of social bees (Koch and Schmid‐Hempel 2011, Koch et al. 2013; Martinson et al. 2011; Powell et al. 2014).

However, the impact of social behavior on the ecological and evolutionary dynamics of the bee microbiome remains poorly understood, with a range of recent studies suggesting a more complex picture. For example, some lineages of the highly eusocial Meliponini appear to have entirely lost members of the “corbiculate core” microbiome (Koch et al. 2013; Cerqueira et al. 2021; Kueneman et al. 2023), and even within the same bumblebee species, individuals may frequently contain “disrupted” microbiomes lacking the “corbiculate core” bacteria and instead predominantly harbor environmental bacteria (Li et al. 2015; Villabona et al. 2023; Hotchkiss et al. 2025). Kueneman et al. (2023) suggested that tongue length (reflecting different foraging behaviors rather than social behavior) may be a better determining factor for gut microbiomes in different species of the socially polymorphic orchid bees (Euglossini). In halictid bees, no strong impact of social behavior on microbiome composition has been found across *Lasioglossum* species with solitary or social behavior (Rubin et al. 2018), or within *Megalopta* species with solitary or social colonies (McFrederick et al. 2014). On the other hand, species of socially polymorphic *Xylocopa* consistently exhibit gut microbiomes closely related to “corbiculate core” bacterial taxa (Holley et al. 2022; Handy et al. 2023; Gu et al. 2023). Hence, there are no clear patterns as to why the gut microbiomes of some socially polymorphic bees show affinities with the core microbiome of corbiculate bees, while others do not. An environmentally controlled assessment of microbiomes among the tribes of Xylocopinae with sympatric corbiculate bees might advance our knowledge in this regard (Box 1).

The microbiomes of bee larvae are not as well sampled as for adults, but larvae do consistently show distinct differences from adults across different species and levels of sociality and therefore deserve separate attention (McFrederick et al. 2014; Parmentier et al. 2018; Kapheim et al. 2021; Kowallik and Mikheyev 2021). In honeybees, guts of healthy larvae contain variable communities of bacteria and lack the “core” adult honeybee lineages (Martinson et al. 2012; Kowallik and Mikheyev 2021). Some acetic acid bacteria (*Bombella apis* = syn. *Parasaccharibacter apium*) and lactic acid bacteria (e.g., *Lactobacillus kunkeei* ) are, however, frequently found in larvae (Vojvodic et al. 2013; Corby‐Harris et al. 2014; Smith et al. 2021; Kowallik and Mikheyev 2021) and can have beneficial effects against fungal and bacterial larval pathogens (Arredondo et al. 2018; Miller et al. 2021). Gut microbiomes of honeybee larvae are “decoupled” from adult gut microbiomes due to the loss of the larval microbiome in the pupal stage and a recolonization of microbiota in newly emerged adult bees (Powell et al. 2014; Kowallik and Mikheyev 2021); possibly a general feature across many bees, all of which undergo holometabolous development (Koch and Schmid‐Hempel 2011; Hammer and Moran 2019).

Among solitary mass provisioning bees, distinctions between microbiomes of different ontological stages may be driven by temporal and environmental exposure to different diets: because larval microbiomes resemble predominantly pollen‐based brood provisions, especially in the early instars (Kapheim et al. 2021; Nguyen and Rehan 2022); whereas adult gut microorganisms frequently derive from nectar or contact with flower surfaces (McFrederick et al. 2012; Li et al. 2023). However, in social species, close contact with (and feeding of) larvae by adults can transmit microorganisms back and forth between adults and larvae (Folly et al. 2017), and differences in adult and larval microbiomes may be more directly linked to physiological and physicochemical differences in the gut environment across the two life stages selecting for different colonizing species (Hammer and Moran 2019). We might expect allodapine bees to show lesser degrees of differentiation between ontological life stages given: (1) the progressive nature of brood provisioning which would dilute temporal differences in floral availability (*cf*. mass prvisioning bees); as well as (2) adult handling of larvae and frequent removal of their fecal pellets.

## 5 Microbial Associates of Allodapine Bees

### 5.1 Literature Review

We undertook a survey of primary literature detailing microbiota of allodapine bees within *Web of Science Core Collection* (Clarivate Analytics—accessed January 2025) using default Document search options within the “Topic” field. We searched taxonomic terms [allodapin*; *Allodape*; *Allodapula*; *Braunsapis*; *Brevineura*; *Compsomelissa*; *Effractapis*; *Eucondylops*; *Exoneura*, *Exoneurella*; *Exoneuridia*; *Hasinamelissa*; *Inquilina*; *Macrogalea*; *Nasutapis*; *Ceratina*; *Xylocopa*; *Manuelia*; *Bombus*; *Apis*] in combination with research terms [bacteria; fungi; immunity; microbiome; microbiota; microsporidia; *Nosema*; pathogen; pollinat*; virus]. Publication matches (“hits”) were scored as “raw‐hits” as a first‐pass. Within Xylocopinae, articles were read and “verified” to confirm that research pertained to the search terms, rather than referential mentions in the text. Second‐pass “verified‐hits” were also tallied.

Literature search outcomes (Table 2), indicate relatively limited microbial research on allodapine bees. Topic filters “bacteria”, “fungi”, and “pathogen” only captured one paper—a project exploring antimicrobial activity of allodapine, anthophorine and meliponine bees in response to pathogenic fungi (Stow et al. 2010). The remaining search terms were unavailing. Some results represent false flags, wherein the search filters identified papers that contained matching species nomen of mushrooms (inquilina + fungi) and hydroids (inquilina + pathogen); or bee research papers which mentioned our target fields (pathogen, pollinat*) but undertook no research on those respective topics. Verified hits to target bees and research topics are provided in parentheses for Allodapini, Ceratinini and Xylocopini (Table 2). Over the last decade, a flurry of microbial research has been undertaken on the sister tribe Ceratinini, whereas studies on Xylocopini have been sporadic since the 1990's. There have been no studies on Manueliini.

**TABLE 2:** Literature search.

	Web of Science	Bacteria	Fungi	Immunity	Microbiome	Microbiota	Microsporidia	Nosema	Pathogen	Pollinat*	Virus
Apidae											
Xylocopinae											
Allodapini											
Allodap*	176	0	0	0	0	0	0	0	2 (0)	29 (8)	0
*Allodape*	10	0	0	0	0	0	0	0	0	4 (4)	0
*Allodpaula*	9	0	0	0	0	0	0	0	0	3 (3)	0
*Braunsapis*	48	0	0	0	0	0	0	0	1 (0)	20 (9)	0
*Brevineura*	7	0	0	0	0	0	0	0	0	1 (0)	0
*Compsomelissa*	5	0	0	0	0	0	0	0	0	1	0
*Effractapis*	0	0	0	0	0	0	0	0	0	0	0
*Eucondylops*	0	0	0	0	0	0	0	0	0	0	0
*Exoneura*	72	1 (1)	1 (1)	0	0	0	0	0	1 (1)	11 (9)	0
*Exoneurella*	14	1 (1)	1 (1)	0	0	0	0	0	1 (1)	1 (0)	0
*Exoneuridia*	0	0	0	0	0	0	0	0	0	0	0
*Hasinamelissa*	1	0	0	0	0	0	0	0	0	0	0
*Inquilina*	72	0	2 (0)	0	0	0	0	0	1 (0)	2 (0)	0
*Macrogalea*	14	0	0	0	0	0	0	0	0	0	0
*Nasutapis*	1	0	0	0	0	0	0	0	0	0	0
Ceratinini											
*Ceratina*	310	8 (7)	8 (6)	3 (3)	12 (11)	6 (6)	0	0	7 (5)	108	2 (2)
Xylocopini											
*Xylocopa*	710	11 (10)	8 (3)	1 (1)	6 (6)	5 (5)	0	2 (2)	11 (9)	399	6 (4)
Manueliini											
*Manuelia*	11	0	0	0	0	0	0	0	0	1 (1)	0
Apinae											
Bombini											
*Bombus*	5578	125	64	118	49	100	79	199	453	3072	162
Apini											
*Apis*	36,172	1199	517	814	229	480	396	1400	2068	4947	1648

*Note:* Number of Web of Science publications matching target microbial and pollination search terms (blue columns) for all genera of Xylocopinae and select genera of Apinae. Raw hits listed for all taxa with verified content in parentheses for Xylocopinae (excluding ‘Pollinat*’ for *Ceratina* and *Xylocopa*).

Only three studies have investigated allodapine microbial associates. The first (Stow et al. 2010) explored antimicrobial responses to spore germination and hyphal growth of the entomopathogenic fungus *Beauveria* (syn. = *Cordyceps*) *bassiana*, isolated from *Exoneura robusta* ; subsequently tested on this and another species of *Exoneura* ( *E. nigrescens* ) and one species of *Exoneurella* ( *E. tridentata* ). The second study (Brettell et al. 2020) used multiplexed and real‐time PCR methods to screen well known honey bee fungal microsporidian parasites (*Nosema*) and RNA viruses (black queen cell, Israeli acute paralysis, Lake Sinai, sacbrood) within seven *Exoneura* specimens: likely, *E. robusta* and *E. angophorae* based on parallel studies at the same locality (Blue Mountains, New South Wales: Bernauer, Tierney, et al. 2022; Bernauer, Cook, et al. 2022; Bernauer et al. 2024; Tierney et al. 2023). *Exoneura* specimens tested positive for Black queen cell virus, Lake Sinai virus 1 and 2, and Sacbrood virus, albeit at low prevalence. The third study (Mee and Barribeau 2023) screened published transcriptome libraries of bees for microbial associates and included a library from the *Exoneura* sp. in Brettell et al. (2020). Mee and Barribeau (2023) failed to detect bacteria in the transcriptome with their analysis pipeline but reported on the presence of the fungi/Microsporidia *Alternaria*, *Aspergillus*, *Aureobasidium*, *Candida*, *Colletotrichum*, *Nosema*, *Penicillium*, and *Starmerella*. Except for the known bee‐parasitic microsporidian genus *Nosema*, these fungi are all common environmental fungi often associated with pollen and nectar (Mee and Barribeau 2023); likely present in the gut of the bee from a dietary source.

The most common microbial associates of Ceratinini and Xylocopini (Table S2) provide an informative guide for future studies of Allodapini. Dominant bacterial groups derive from: (1) brood food mass provisions (pollen & nectar); and (2) gut microbiomes of adults and immatures (larvae, pupae). For ceratinines, there are patterns of declining bacterial diversity with progressive developmental stages, with adults exhibiting the lowest diversity; but no differences in fungal diversity across developmental phases (Nguyen and Rehan 2022). Furthermore, the pollen diet breadth of *Ceratina* mass provisions has been shown to influence fungal diversity, but not bacterial diversity (Gaiarsa et al. 2022); and there are general patterns of diet breadth (as well as bacterial and fungal diversity in mass provisions) negatively correlating with latitude (McFrederick and Rehan 2019). Bacterial components of *Xylocopa* microbiomes show evidence of shared and unique genera (Table S2), while less attention has been given to fungal components (*cf*. *Ceratina*) beyond entomopathogenic fungi causing chalkbrood (*Ascosphaera*) and microsporidian parasites (*Nosema*). However, there has been more research undertaken on the spillover of DNA and RNA viruses common to honey and bumble bees to *Xylocopa*, compared with other tribes; and *Xylocopa* is the only group within Xylocopinae with reported infections of deleterious protists (*Apicystis*, *Crithidia*).

### 5.2 Cobiont Genome Screen

Genome sequencing projects can inadvertently sequence organisms closely associated with the target host, such as bacteria or parasitic eukaryotes (“cobionts” *per* Vancaester and Blaxter 2023), and thereby discover novel members of the host microbiome (e.g., Martinson et al. 2014). Transcriptome data has previously been investigated for microbial associates of one allodapine species (Mee and Barribeau 2023), and here we focus on published whole genome sequence data. A search for published genome sequencing projects of allodapine bees on GenBank found draft genomes for *Exoneura robusta* (GCA_019453415.1) and *Exoneurella tridentata* (GCA_019453975.1). We used the BlobToolKit viewer (Challis et al. 2020) for each genome to detect contigs assigned to phyla other than Arthropoda and downloaded all non‐Arthropoda contigs separately by phylum from GenBank. We then carried out BlastN searches recovering sequences with significant alignment to the reference sequences: *Escherichia coli* reference 16S rRNA gene sequence (NR_024570.1) against Proteobacteria contigs; *Nosema apis* reference small subunit ribosomal ribonucleic acid (SSU rRNA) sequence (U26534) against Microsporidia contigs; and *Apicystis bombi* reference sequence (FN546182; Schoonvaere et al. 2020) against gregarine contigs.

Sequences aligning to the reference were subjected to a BlastN search (Altschul et al. 1990) against the NCBI core nucleotide database (core_nt) to find the most similar published sequences. We then aligned the respective sequences to the most similar identified sequences from core_nt using ClustalW 2.1 (Larkin et al. 2007) and included sequences of relevant known bee‐associated microorganisms for each taxon (e.g., *Nosema apis* and *Nosema ceranae* for Microsporidia and bee associated gregarines in Schoonvaere et al. 2020). Where applicable, we aligned the reverse complement of the sequences recovered from genomic contigs. Maximum likelihood phylogenetic trees were constructed with PhyML 3.0 (Guindon et al. 2010), including 100 bootstrap replicates and Bayesian Information Criterion for automatic model selection.

**FIGURE 3:**
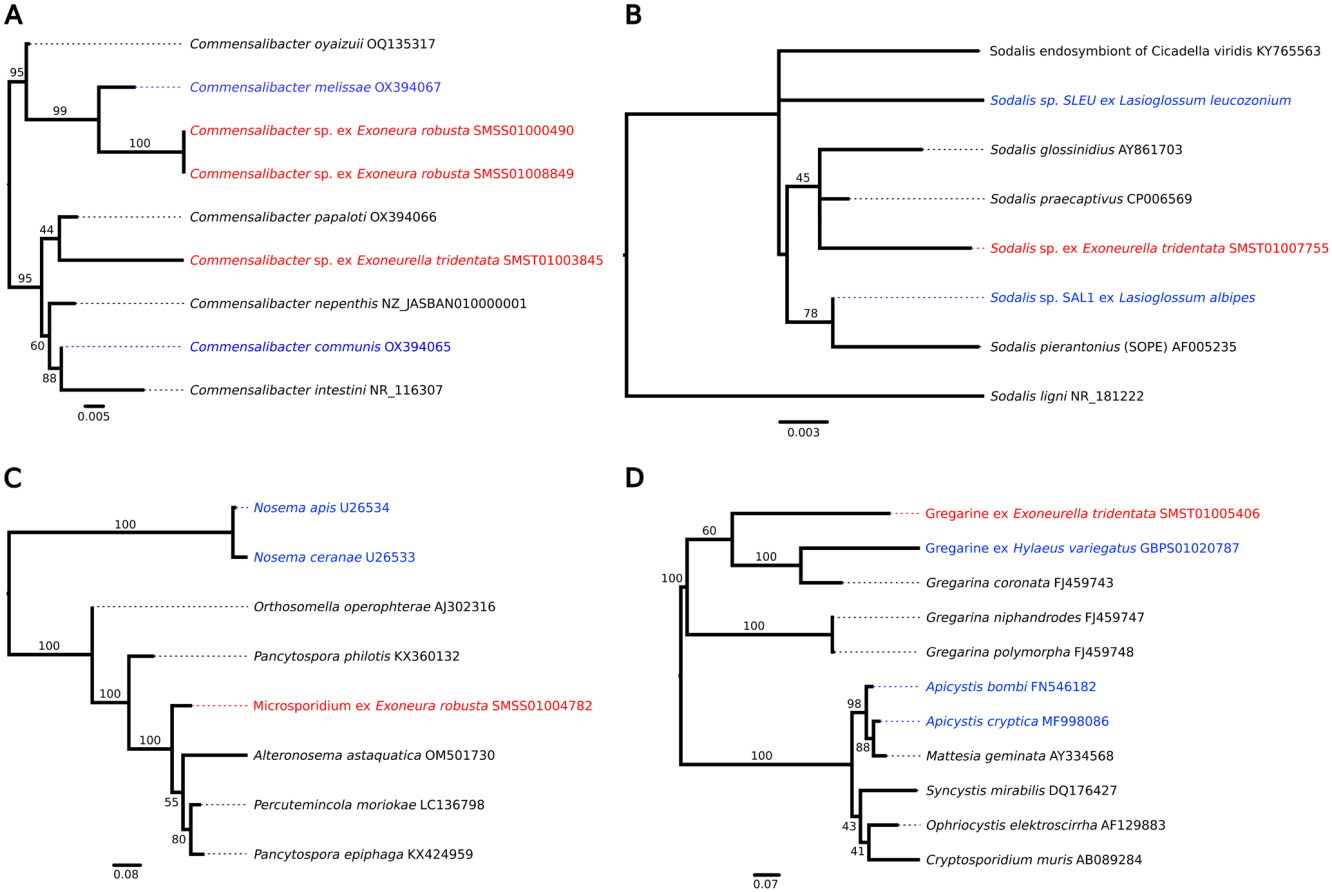
Discovery of cobionts. Maximum likelihood phylogenies of SSU rRNA genes for cobionts of allodapine bees identified within genome sequencing projects, namely: (A) *Commensalibacter* sp.; (B) *Sodalis* sp.; (C) Microsporidia; (D) Gregarines. Tree branch lengths represent nucleotide substitutions per site, with Bootstrap node support placed on the ancestral branch. Red terminal labels: Sequences from allodapine genome sequencing projects. Blue terminal labels: Other bee associated microorganisms. GenBank accession numbers follow terminal branch nomens (except SAL1 & SLEU: From Rubin et al. 2018).

The *Exoneura* genome contained 92 contigs assigned to the phylum Proteobacteria and 30 contigs of Microsporidia, whereas the *Exoneurella* genome had 182 contigs assigned to Proteobacteria. Among the Proteobacteria, both *Exoneura* and *Exoneurella* had a representative of the Acetobacteraceae genus *Commensalibacter* sp. (Figure 3A). The two 16S rRNA gene sequences from *Exoneura* were identical and formed a sister group to the known honeybee associated bacterium *Commensalibacter melissae* (~3% sequence divergence), tentatively suggesting the presence of a novel *Commensalibacter* species in *Exoneura*. The *Commensalibacter* sp. from *Exoneurella* was distinct and within a clade of other insect associated species including *C. papaloti*, *C. nepenthis*, *C. intestini*, and *C. mensalis*. The *Exoneurella* genome further contained a *Sodalis* sp. (Enterobacteriaceae) (Figure 3B) related to known insect‐associated taxa including two strains from *Lasioglossum* bees, as well as an *Acinetobacter* sp. (Moraxellaceae) with 99.45% identity to the type strain of the nectar bacterium *Acinetobacter nectaris* (NR_118408; Álvarez‐Pérez et al. 2013) and a *Pseudomonas* sp. (Pseudomonadaceae)—see Table S3.

The SSU rRNA gene of the microsporidium in the *Exoneura* genome was distinct from known bee parasites *Nosema apis* and *Nosema ceranae* (Figure 3C), and was most closely related to several sequences from nematode‐associated taxa (*Pancytospora philotis*, *Pancytospora epiphaga*, *Percutemincola moriokae*), and a crayfish‐associated microsporidium (*Alteronosema astaquatica*). The gregarine of *Exoneurella* fell within a clade of other insect‐associated gregarines in the genus *Gregarina* sp. (Figure 3D) including a sequence from an undescribed species derived from a *Hylaeus* colletid bee host, but distinct from known bee gregarine taxa *Apicystis bombi* and *Apicystis cryptica*.

### 5.3 Detection of Novel Cobionts

Our screening of microbial associates in published genomes of allodapines adds several novel microbial taxa not previously recorded in this group of bees (highlighted blue in Table S2). The most notable of these bacterial (*Commensalibacter*, *Sodalis*, *Acinetobacter*) and microsporidian parasite discoveries are discussed below.

*Commensalibacter* species were present in both *Exoneura robusta* and *Exoneura tridentata* that are distinct from previously described insect‐associated taxa (Botero et al. 2023), suggesting novel diversity of this group of bacteria in allodapine bees. *Commensalibacter* has only occasionally been found in Xylocopini (Holley et al. 2022; Gu et al. 2023) indicating it is not part of a stable core microbiota across subfamily Xylocopinae (see Section 7 for a précis of bacteria in *Xylocopa* that resemble corbiculate bee core‐microbiomes); and some *Commensalibacter* species have been found in a range of different flower‐visiting insects (e.g., *C. communis* —Botero et al. 2023) suggesting environmental transmission. However, the *Commensalibacter* most closely related to that found here in *Exoneura* ( *C. melissae* ) appears to be exclusively specialized on honey bee hosts. Further work will be needed to clarify whether the *Commensalibacter* in allodapines comprise specialized lineages inhabiting the allodapine gut or are generalists that are extrinsically shared between the local pollinator community.

The presence of *Sodalis* sp. bacteria in *Exoneurella tridentata* adds allodapines to the list of known bee hosts, in addition to previous records for *Ceratina* (see Table S2) and halictid bees (Rubin et al. 2018). Related species of *Sodalis* represent both obligate intracellular endosymbionts and free living lineages within a range of insect hosts, including Coleoptera, Diptera, Hemiptera, Hymenoptera, and Psocodea (see Rubin et al. 2018 and references therein). Enterobacterales deserve further study in allodapines in order to improve our comparative understanding of their functional impact on bee hosts and their potential links to social evolution (Box 1). The bacterial gut microbiota of newly emerged adult honey bees is known to be influenced by the presence of social worker castes (nurse bees—Powell et al. 2014). Whereas, a comparison of *Sodalis* abundance among mass provisioning halictine bees yielded equivocal results (Rubin et al. 2018) among solitary species, obligately social species and facultatively social species of *Lasioglossum*—namely, three *Sodalis* strains were more prevalent among solitary bees while a different strain was in higher abundance in social bees. Rubin et al. (2018) speculated that social behaviors may be responsible for the restriction of *Sodalis* among social halictine bees across the phylogeny, in a similar manner to group‐based behavioral responses to pathogens—termed “social immunity” (see Section 6).

The *Acinetobacter* we found in *Exoneurella* belonged to a group of nectar‐inhabiting species that is frequently found in bee guts (Sanchez et al. 2024), and likely reflects part of the bees' bacterial community that is environmentally acquired via foraged nectar and pollen. Notably, members of the specialized “corbiculate core” bacterial gut microbiome (see above) were absent both here and in the analysis of *Exoneura* transcriptomic data by Mee and Barribeau (2023), but clearly more detailed sampling across the tribal phylogeny is needed to verify whether these bacterial lineages are consistently absent or rare among allodapines.

The novel microsporidian and gregarine parasite sequences we recovered from *Exoneura robusta* and *Exoneurella* may represent parasite lineages previously unknown among bees. The close link to nematode‐affiliated species of microsporidium (*Pancytospora*, *Percutemincola*) found here in *Exoneura* (Figure 3C), alternatively suggests derivation from a nematode host—a parasite group known to infect allodapines (Bernauer et al. 2021). However, no nematode‐related sequences were found in the *Exoneura* genome dataset, and the related *Alteronosema astaquatica* infects an arthropod (crayfish), leaving the possibility of this being a genuine parasite of *Exoneura*. Very few specialized eukaryotic parasites are known from bees outside of the highly eusocial honey and bumble bees (Engel et al. 2016), although whether this reflects the clear research bias toward corbiculate bees (see Table 2) or a genuine rarity of parasites evolving host specialization in bees without eusocial behavior (as may be expected from theory, Schmid‐Hempel 2021), remains to be determined.

Our finding of several lineages of novel bacteria and parasitic eukaryotes (in even a very small sample of just two allodapines genome sequencing projects) hints at the possibility that allodapines (and their facultative social organization) may be a rewarding system to explore associations between host specialization and sociality for both the bacterial microbiome and microbial pathogens. As illustrated here, the “by‐catch” of cobionts from genome sequencing projects are likely to provide a valuable resource to discover new microbial associates of allodapine bees.

## 6 Social Immunity

The primary motivation for previous research on allodapine microbiota (Table S2) derives from whether microbial pathogens may have driven the evolution of group living and social organization, particularly for defence against pathogenic fungi (Stow et al. 2007, 2010). The theoretical expectation that social organisms should exhibit heightened defence mechanisms to cope with parasites and associated pathogens (defined as a collective or “social immune system” Cremer et al. 2007), is based on the rationale that there is an increased risk of disease transmission inherent to group living. Risks should be even more acute among haplodiploid social insects colonies composed of genetically similar individuals (due to kin selection) that are frequently interacting with one another. This close spatial and genetic distance within social insect colonies facilitates the spread of parasites (Schmid‐Hempel 2021), aspects that may be further elevated in the case of allodapine linear stem nest architecture and progressive brood rearing in the absence of brood cell divisions (*cf*. mass provisioning stem and ground nesting bees that do not physically interact with immature brood).

Selective evolutionary processes promoting immunity are likely to have operated differently at individual versus colonial levels, and whether social immune systems represent an evolutionary elaboration or regression of pre‐existing immune responses found in ancestral solitary insect lineages is up for debate given conflicting empirical evidence (*reviewed by* van Meyel et al. 2018). Our literature review (Table 2) indicates that the majority of research has been conducted on economically important bee pollinators (*Apis*, *Bombus*). However, for some time there has been recognition of the need to explore the characteristics of collective immune responses among smaller, less complex social insect lineages (Cremer et al. 2007)—such as those exemplified by the Xylocopinae.

Stow et al. (2007) tested antimicrobial activity of body surface extracts against a non‐specific pathogenic bacterium ( *Staphylococcus aureus* ) across six bee species with varying levels of social organization (solitary, semi‐social, and eusocial), including three allodapine species (two *Exoneura* and one *Exoneurella*). Antimicrobial activity increased from solitary to eusocial species, supporting a link between higher levels of sociality and immune defence. However, both representatives of solitary (single‐female) bees and semi‐social bees were each from the same genus, resulting in limited phylogenetically independent contrasts (Felsenstein 1985) that was not considered in the analysis and place reservations on the strength of the result. Measuring aspects of the “internal” immune defence system of bees of varying levels of sociality like antimicrobial peptide expression would provide an independent complement to Stow et al.'s (2007) approach in measuring the external immune defence. Furthermore, the various bee taxa under comparative investigation: (a) inhabit very different climatic zones (semi‐arid, temperate heathlands, wet montane forest, tropics) from one another; (b) construct nests of very different architecture and nesting substrates (ground, aerial plant stems, tree cavities); and (c) both *Exoneura* species are capable of forming ontogenetic eusocial colonies (i.e., *Exoneurella* is sometimes mistakenly classified as the only allodapine genus that forms eusocial colonies).

An intraspecific test of Social Immunity in the facultatively social *Ceratina okinawana* , Nguyen et al. (2023) found that group size was influential in determining an individual bee's antimicrobial efficacy. This supports the “Eusocial Framework” (Cremer et al. 2007) – that social immunity is a secondary trait that arose after the advent of collective nesting, as opposed to an ancestral trait that promoted the evolution of collective nesting (per ‘Group Living framework’ Munier 2015). A broader definition of the Eusocial Framework may be warranted (Cremer et al. 2018; van Meyel et al. 2018), given that *C. okinawana* is not eusocial and Social Immunity has previously been demonstrated in subsocial earwigs (Diehl et al. 2015) and beetles (Reavey et al. 2014). Hence, density dependent prophylactic effects (group size) may be more important in the evolution of Social Immunity than social organization per se. Similar to halictine bees (Schwarz et al. 2007), allodapines would provide many avenues to explore such matters in greater manipulative experimental detail given the phylogenetic diversity and range in social organization as ontogenetic stages of colony maturity (Michener 2007 p. 13). Because as mentioned above, unless nests are co‐founded, most allodapine colonies begin as subsocial assemblages that either develop into casteless colonies (equivalent to communal halictine nests—see Dew et al. 2016) or semisocial and eusocial colonies (due to frequent overlap of brood generations).

## 7 Comparative Microbial Assemblages

The distinctive and highly conserved core gut microbiome profiles of eusocial corbiculate bees (e.g., Martinson et al. 2011) have led to preconceptions that socially transmitted core microbiota are important for maintaining the health of large colonies; whereas solitary bee microbiota are presumed to be considerably more variable owing to the complete reliance on environmental acquisition of microbiota and lack of nestmate interactions, especially the lessened degree of vertical transmission (McFrederick et al. 2017; Voulgari‐Kokota, Ankenbrand, et al. 2019; Voulgari‐Kokota, McFrederick, et al. 2019). These perceptions may simply be an artifact of the dearth of comparative evidence and have subsequently been tempered by the discovery of very consistent bacterial microbiomes across four species of *Xylocopa* that mirror the core bacterial microbiota found in honey and bumble bees in North America (Holley et al. 2022; Handy et al. 2023) and Asia (Gu et al. 2023). There was also a lack of intraspecific microbial differentiation between solitary and social nests of facultatively social augochlorine halictid bees (McFrederick et al. 2014).

Another important distinction, is that honey and bumble bees also engage in progressive provisioning of brood, where adults can influence the bacterial microbiome of larvae. Whereas in mass provisioning bees, an early larval instar's microbiome is expected to be predominantly influenced by the bacterial composition of the pollen/nectar food provision (McFrederick et al. 2014; Dew et al. 2020; McFrederick and Rehan 2019). Therefore, we might expect the microbiome profiles of the progressively provisioning allodapine bees to parallel the conserved nature of *Apis* and *Bombus* ‘core‐microbiomes’ (Martinson et al. 2011), more so than their closest phylogenetic relatives *Ceratina* and *Xylocopa* which mass provision brood cells—see Figure 2. However, experimental evidence (Powell et al. 2014) suggests that adult‐adult trophallaxis is not a sufficient mechanism to transmit the core microbiome from a mature worker to a newly emerged worker, presumably because the bumble and honey bee microbiome is mostly located in the hindgut, whereas the crop (source for regurgitated trophallaxis) may not have much of a resident‐core microbiome. Hence, a faecal‐oral transfer route may be more important mode of microbial transmission contributing to the core microbiome of corbiculate bees, because bumble bees do not engage in trophallaxis and callow adults acquire gut microbes from their mature adult sisters' faecal pellets in the bottom of the nest (Koch and Schmid‐Hempel 2011).

One key goal for understanding the bee microbiome lies in progressing from describing patterns of microbiome variation (within and between species) to understanding the underlying functional ecology and evolutionary processes involved. On the ecological side, differences in bee behavior such as in floral preferences and social behavior will expose bees to different external pools of microbes, that when ingested, will result in variations in either transient or colonizing microorganisms in the bee gut. Some of the observed differences in wild bee microbiomes could be explained purely by such ecological processes, especially if associations are mainly with transient environmental microorganisms encountered at floral hubs (Kueneman et al. 2023). Priority effects (order of arrival), likely play an important role in shaping an individual's microbiome (Debray et al. 2022); for example, in social bees, colonization of a newly emerged bee by microbes from nestmates can prevent colonization by other microbial species or strains. A social environment can thus facilitate colonization with benign or beneficial microbes that provide a ‘future‐proof’ colony‐level resistance against subsequent interactions with pathogens (Koch and Schmid‐Hempel 2011), and hosts may exhibit behaviors that actively facilitate the uptake of beneficial microbes (Foster et al. 2017). The host can play a further active role in influencing the ecological processes in the gut microbiome, by “managing” the gut microbiome as an “ecosystem on a leash” (Foster et al. 2017), through its immune system.

This effectively represents the creation of evolutionary selective environments via manipulation of the physicochemical niche or by providing specific nutrients that encourage colonization by beneficial symbionts (Foster et al. 2017; Quinn et al. 2024). Host microbiome “management” mechanisms may confer selective advantages (e.g., reducing pathogen infection loads or facilitating digestion of the diet) that could be subject to natural selection. Different bee species might be expected to develop different strategies to “manage” their microbiome depending on factors such as their dietary preferences, pathogen pressures, or social behavior, and strategies may range from entirely preventing microbiome colonization (Hammer et al. 2019) to establishing very selective associations with individual microbial lineages (Kwong et al. 2017; Quinn et al. 2024). From a microbial perspective, adaptation to the environment of specific hosts at evolutionary scales might require consistent transmission between host generations, which is likely facilitated by social interactions between mothers and daughters in bees—vertical transmission. If microbial transmission is predominantly vertical, the reproductive success of microbes and hosts is aligned, and selection may favor beneficial microbial strains (Koch and Schmid‐Hempel 2011).

A second, more applied goal for bee microbiome research lies in better understanding the functional importance of the microbiome of wild bees, and the influence that human management interventions can play on microbiome function (Box 1). Anthropogenic influences like changes in floral landscapes or pollutants may disrupt healthy microbiomes in bees (Motta et al. 2018; Koch et al. 2019). Alternatively, deliberate probiotics interventions (Motta et al. 2022; Nguyen and Rehan 2025) or augmenting floral landscapes with key plant species to help prevent parasite infections could protect wild bees against anthropogenic stressors (Stevenson et al. 2022; Malfi et al. 2023).

Allodapine bees, again, present an intermediate natural history point of difference in this respect. Progressive provisioning of larvae in a linear tunnel with no brood cells means that transmission pathways are likely different from most mass‐provisioning solitary bees, because there is enhanced potential for disease and symbiont transmission to/from larvae through alloparental rearing by multiple adult nestmates. Modes of direct adult‐larva and adult‐adult contact in nests, especially via trophallaxis and removal of fecal pellets, may facilitate transmission and evolution of specialized microbial lineages that are more similar to honey and bumble bees. The intraspecific plasticity of social organization in some exoneurine nests, containing one to several females, combined with broad geographic ranges spanning different climates along altitudinal and latitudinal gradients (e.g., Neville et al. 1998; Tierney et al. 1997; Steen and Schwarz 1998; Cronin and Schwarz 1999, 2001; Cronin 2001; Joyce and Schwarz 2006; Dew et al. 2018a, 2018b; Bernauer et al. 2021), means that allodapines could provide an ideal model group to study the impact of social interactions and ecological factors on the evolution of host microbiomes. Hence, allodapine bees may enable insights for the kind of integrated understanding of socio‐eco‐evo‐immunology envisaged by Cremer et al. (2018).

There is an applied need to fill knowledge gaps on the threats or benefits from microbial associates of allodapine bees (given their important role in providing pollination services—see Section 2 above), beyond their potential as a model system to study the role of sociality in the ecology and evolution of bee microbiomes. Allodapines may, for example, possess specialized microbial symbionts like other social bees (see Motta and Moran 2024) that could play roles in digesting and detoxifying their diets, or in pathogen defense.

## 8 Disease and Pollination Services

The effect of parasites and associated pathogens on bee foraging and pollination has been reviewed by Koch et al. (2017). Horizontal transmission of many parasites occurs while bees are foraging for pollen and nectar on flowers, underlining the importance of understanding the basic natural history of which flowers different bee species visit and the functional role of the respective plant traits (e.g., floral architecture, McArt et al. 2014). Such information is crucial for attempts to investigate and control bee disease, because which flower species bees visit influences transmission and immune responses. Honey bee pathogens including viruses and Microsporidia can spill over from managed or invasive honeybee colonies to wild pollinators on shared flowers (Fürst et al. 2014; Graystock et al. 2015; Koch et al. 2017; Purkiss and Lach 2019), but impacts of this on wild pollinator populations are still poorly understood.

Pollen and nectar chemistry can promote or inhibit parasitic driven pathogens and significantly alter bee nutritional health, immune responses, and microbiome profiles. Researchers are beginning to explore whether bees possess an adaptive ability to reduce the risk of disease exposure at flowers via behavioral strategies that spatio‐temporally partition foraging at shared floral resources (reviewed by Nicholls et al. 2022); provided this can be divorced from the selective pressure of resource competition alone. Ultimately, poor bee health has deleterious effects on pollination services by reducing population sizes, which is significant given that pollinator visitation abundance is the primary parameter influencing pollination efficacy (Danforth et al. 2019; Tierney et al. 2023). Furthermore, bumble and honey bees infected by protozoans (*Nosema*, *Crithidia*) have been shown to exhibit sub‐optimal foraging behavior resulting in reduced plant reproduction or alternate floral preferences linked to infection rates (*see* Koch et al. 2017 and references therein).

From an allodapine‐centric perspective, this is important for both human food security (pollination services Table S1) as well as ecological function in natural landscapes. These issues will become acutely apparent should horizontal transfer of diseases from managed pollinators to native bees become prolific in the future—as alluded to for *Varroa‐transmitted* diseases such as deformed wing virus in Australia (Chapman et al. 2023). There is considerable disparity in the quantity of pollination research conducted on allodapines, as indicated in Table 2 (verified hits: 9 studies on *Braunsapis* mainly in Asia; 9 on *Exoneura* in Australia; 4 on *Allodape* and 3 on *Allodapula* in Africa), compared with the other apid genera investigated in this review (unverified hits: 108 on *Ceratina*, 399 on *Xylocopa*, 3072 on *Bombus* and 4947 on *Apis* globally).

In natural landscapes, Allodapine bees have been reported visiting the flowers of 50 angiosperm genera from 30 families (Table S4), by researchers working in Africa (Burkina Faso, Gabon, South Africa), Asia (India) and Australia. Australia has the broadest sampling of allodapines in natural landscapes (especially for Western Australia—Houston 2000), where endemic exoneurine bees (*Exoneura*, *Brevineura*, *Exoneurella*) commonly visit flowers of Myrtaceae (*Eucalyptus*, *Melaleuca*), Papilonaceae, Proteaceae (*Dryandra*, *Grevillia*), Myoporaceae (*Eremophila*) and Goodeniaceae (*Scaevola*).

## 9 Conclusions

Our review elucidates several key aspects pertaining to the microbial evolution of allodapine bees in comparison with related tribes within Xylocopinae (Ceratinini, Xylocopini) and corbiculate bees within the related subfamily Apinae (Apini, Bombini). The unusual nesting biology of allodapines facilitates network trophallaxis among nestmates that we predict is likely to manifest in a “network microbiome”. This may result in a relatively regimented microbial profile– in parallel to those of highly organized and demographically much larger eusocial bee colonies with constrained vertical transmission of microbiota (*Apis*, *Bombus*)—more so than the allodapines' closest phylogenetic relatives (*Ceratina*, *Xylocopa*).

Despite being frequent visitors to and pollinators of crops and wild plants in Africa, Asia and Australia, knowledge of allodapine microbiomes and microbial pathogens remain relatively obscure. We find hitherto unknown eukaryotic microsporidian and protozoan parasites in published genomic data of allodapine bees, as well as members of the known bee or insect associated bacterial genera (*Commensalibacter*, *Sodalis*). However, among allodapine bees studied thus far, there is no evidence of typical “corbiculate core gut microbiome” bacteria (*Snodgrasella*, *Gilliamella*) or bee‐specific Lactobacilli or Bifidobacteria.

The intra‐and interspecific diversity in allodapine social organization and their unique progressive larval rearing modes would make them attractive model species to study the link between sociality, immunity, and the microbiome at colonial, population, ecological, and evolutionary scales. Given the cumulative evidence of pathogen transmission from managed and feral honey bees to wild bees, the risk to native allodapine bees from spillover pathogens (e.g., deformed wing virus; Microsporidia) deserves to be comprehensively evaluated, especially due to the undocumented and underappreciated pollination services that native bees provide to agroecology and natural landscape ecology globally.

## Ethics Statement

The authors confirm that this manuscript has not been submitted elsewhere, and all research meets the ethical guidelines of Australia.

## Consent

The authors have nothing to report.

## Conflicts of Interest

The authors declare no conflicts of interest.

## Supporting information


Data S1.

## Data Availability

Data for this study are available in the Supporting Information (Tables S1–S4) and Western Sydney University research data store: Tierney et al. (2025) (https://doi.org/10.26183/nhm3‐jh91).
