# Supplementary material for: Microbial Evolution in Allodapine Bees: Perspectives From Trophallactic, Socially Plastic Pollinators
Source: Evol Appl. 2025 Jul 27;18(7):e70137. doi: 10.1111/eva.70137 (PMC12301265; doi:10.1111/eva.70137)
Supplement: Supplementary file 1 — Data S1. [file EVA-18-e70137-s001.docx]

**Electronic Supplementary Material**

**Microbial evolution in allodapine bees:**

**perspectives from trophallactic, socially plastic pollinators**

**Contents**

**Table S1. Allodapine bee visitation to food crops…………………..……………………………page 2**

**Table S2. Identified microorganisms within tribes of Xylocopinae…………………………page 3**

**Table S3. Cobiont screens and matches……………………………………………………………page 5**

**Table S4. Angiosperms visited by allodapine bees in natural landscapes ………………page 6**

**References………………………………………….…………………………………………………………page 7**

**Table S1. Allodapine bee visitation to food crops**

| **Bee Genus** | **Food Crop** | **Crop Group** | **Citation** |
| --- | --- | --- | --- |
|  |  |  |  |
| *Braunsapis* | Alfalfa | Leafy vegetables | 1 |
|  | Apple | Pome Fruits | 1 |
|  | Bamboo | Stalk and stem vegetables | 2,3 |
|  | Carrot | Root and tuber vegetables | 4,5 |
|  | Cashew | Tree nuts | 6-8 |
|  | Mango | Tropical fruits | 9,10 |
|  | Muskmelon | Fruiting vegetables, Cucurbits | 9 |
|  | Mustard | Oilseed & Leafy vegetables | 9 |
|  | Sponge gourds | Fruiting vegetables, Cucurbits | 9 |
|  |  |  |  |
| *Compsomelissa* | Cashew | Tree nuts | 6 |
|  | Shea | Oilseed | 11 |
|  |  |  |  |
| *Exoneura* | Apple | Pome Fruits | 12-19 |
|  | Blackberry | Berries and other small fruits | 20 |
|  | Raspberry | Berries and other small fruits | 20 |
|  | Strawberry | Berries and other small fruits | 21 |
|  |  |  |  |
| *Brevineura* | Apple | Pome Fruits | 16-17 |
|  |  |  |  |

**Table S1. Allodapine bee visitation to food crops**. Crop groups derive from the *Codex Classification of Foods and Animal Feeds* (Food and Agriculture Organization 1993). Cited references: 1. Batra 1997; 2. Koshy et al. 2001; 3. Koshy et al. 2022; 4. Batra 1967; 5. Batra 1976; 6. Aidoo 2008; 7. Vanitha & Raviprasad 2019; 8. Kaliaperumal et al. 2023; 9. Batra 1977; 10. Singh et al. 2024; 11. Lassen et al. 2018; 12. Brown et al. 2020; 13. Brown et al. 2022. 14. Bernauer et al. 2021; 15. Bernauer et al. 2022a; 16. Bernauer et al. 2022b; 17. Bernauer et al. 2024; 18. Tierney et al. 2023; 19. Prendergast et al. 2021; 20. Coates et al. 2022; 21. Jaboor et al. 2022.

**Table S2. Identified microorganisms within tribes of Xylocopinae**

| Apidae: Xylocopinae: | Allodapini |  | Ceratinini | Xylocopini |  |
| --- | --- | --- | --- | --- | --- |
|  | ***Exoneura*** | ***Exoneurella*** | ***Ceratina*** | ***Xylocopa*** | Source |
|  |  |  |  |  |  |
| Dominant bacteria |  |  |  |  |  |
| *Acinetobacter* |  | ⚫ | ⚫ | ⚫ | 3-7,9,11,13,25 |
| *Anaerotignum* |  |  | ⚫ |  | 6 |
| *Apibacter* |  |  |  | ⚫ | 13,17 |
| *Arsenophonus* |  |  | ⚫ |  | 9 |
| *Bifidobacterium* |  |  |  | ⚫ | 11-15,17 |
| *Bombiscardovia* |  |  |  | ⚫ | 11-13 |
| *Carnimonas* |  |  | ⚫ |  | 6 |
| *Clostridium* |  |  | ⚫ |  | 9 |
| *Commensalibacter* | ⚫ | ⚫ |  | ⚫ | 12,25 |
| *Cutibacterium* |  |  | ⚫ |  | 6 |
| *Edwardsiella* |  |  | ⚫ |  | 8 |
| *Entomomonas* |  |  |  | ⚫ | 13 |
| *Erwinia* |  |  | ⚫ |  | 3,9 |
| *Fructobacillus* |  |  |  | ⚫ | 13 |
| *Gilliamella* |  |  |  | ⚫ | 13,15 |
| *Lactobacillus sensu lato* |  |  | ⚫ | ⚫ | 3-5,7,10-15,17 |
| *Leuconostoc* |  |  |  | ⚫ | 12 |
| *Melissococcus* |  |  | ⚫ |  | 6,9 |
| *Nocardia* |  |  | ⚫ |  | 7 |
| *Pantoea* |  |  | ⚫ |  | 6,9 |
| *Pseudomonas* |  | ⚫ | ⚫ | ⚫ | 8,11,17 |
| *Saccharibacter* |  |  | ⚫ | ⚫ | 7,9,11,13 |
| *Schmidhempelia* |  |  | ⚫ | ⚫ | 9,12,13 |
| *Serratia* |  |  | ⚫ |  | 8 |
| *Sodalis* |  | ⚫ | ⚫ |  | 3,4,25 |
| *Sphingomonas* |  |  | ⚫ |  | 3,5,6,9 |
| *Wolbachia* |  |  | ⚫ |  | 3,4 |
| *Xenorhabdus* |  |  |  | ⚫ | 11 |
| *Yersinia* |  |  | ⚫ |  | 9 |
|  |  |  |  |  |  |
| Dominant fungi |  |  |  |  |  |
| *Alternaria* | ⚫ |  | ⚫ |  | 7,9,24 |
| *Aspergillus* | ⚫ |  | ⚫ |  | 8,24 |
| *Aureobasidium* | ⚫ |  | ⚫ |  | 9,24 |
| *Candida* | ⚫ |  |  |  | 24 |
| *Cladosporum* |  |  | ⚫ |  | 9 |
| *Colletotrichum* | ⚫ |  |  |  | 24 |
| *Didymosphaeriea* |  |  | ⚫ |  | 6 |
| *Mycosphaerella* |  |  | ⚫ |  | 6,9 |
| *Paraophiobolus* |  |  | ⚫ |  | 6 |
| *Penicillium* | ⚫ |  | ⚫ |  | 6,7,24 |
| *Phaeoacremonium* |  |  | ⚫ |  | 6,9 |
| *Podosphaera* |  |  | ⚫ |  | 9 |
| *Sawadaea* |  |  | ⚫ |  | 9 |
| *Starmerella* | ⚫ |  |  |  | 24 |
| *Taphrina* |  |  | ⚫ |  | 6,9 |
| *Zygosaccharomyces* |  |  | ⚫ |  | 6,9 |
|  |  |  |  |  |  |
| Entomopathogenic fungi |  |  |  |  |  |
| *Ascosphaera* (Chalkbrood) |  |  | ⚫ | ⚫ | 6-9,16,18 |
| *Beauveria* (syn. *= Cordyceps*) | ⚫ | ⚫ |  |  | 1 |
|  |  |  |  |  |  |
| Microsporidian parasites |  |  |  |  |  |
| *Nosema* | ⚫ |  | ⚫ | ⚫ | 8,17,24 |
| aff. *Pancytospora/Percutemincola* | ⚫ |  |  |  | 25 |
|  |  |  |  |  |  |
| Viruses |  |  |  |  |  |
| *A. mellifera* filamentous [DNA] |  |  |  | ⚫ | 22 |
| Black queen cell [RNA] | ⚫ |  |  | ⚫ | 2,19,23 |
| Chronic bee paralysis [RNA] |  |  |  | ⚫ | 23 |
| Deformed wing [RNA] |  |  | ⚫ | ⚫ | 10,19,20 |
| Lake Sinai [RNA] | ⚫ |  |  |  | 2 |
| Sacbrood [RNA] | ⚫ |  |  | ⚫ | 2, 23 |
|  |  |  |  |  |  |
| Protozoan parasites |  |  |  |  |  |
| *Apicystis* (Gregarinasina) |  |  |  | ⚫ | 21 |
| aff. *Gregarina* (Gregarinasina) |  | ⚫ |  |  | 25 |
| *Crithidia* (Trypanosomatida) |  |  |  | ⚫ | 17 |
|  |  |  |  |  |  |

**Table S2. Identified microorganisms within tribes of Xylocopinae**. The Australian exoneurine bees are the only allodapine clade that has been assessed for microbiota. Blue highlight represents novel findings from the current study. Cited references: 1. Stow et al. 2010; 2. Brettell et al. 2020; 3. Dew et al. 2020; 4. McFrederick & Rehan 2016; 5. Graystock et al. 2017; 6. Nguyen & Rehan 2022a; 7. Nguyen & Rehan 2022b; 8. Chau et al. 2023b; 9. Nguyen & Rehan 2023; 10. Santamaria et al. 2018; 11. Holley et al. 2022; 12. Gu et al. 2023; 13. Handy et al. 2023; 14. Alberoni et al. 2019. 15. Subta et al. 2020; 16. Reynaldi et al. 2015; 17. de Landa et al. 2023; 18. Gilliam et al. 1994; 19. Singh et al. 2010; 20. Lucia et al. 2014; 21. Plischuk et al. 2023; 22. Quintana et al. 2019; 23. Radzevičiūtė et al. 2017; 24. Mee & Barribeau 2023; 25. Tierney et al. 2025.

**Table S3. Cobiont screens and matches.**

|  |  |  | GenBank |  |  |
| --- | --- | --- | --- | --- | --- |
| Microbial taxon | Host species | SSU rRNA contig | Closest Hit | Acccession | Seq. match |
|  |  |  |  |  |  |
| **Bacteria** |  |  |  |  |  |
| *Acinetobacter* sp. | *Exoneurella tridentata* | SMST01005573 | *Acinetobacter nectaris* | NR_118408.1 | 99.45% |
| *Commensalibacter* sp. | *Exoneura robusta* | SMSS01000490 & SMSS01008849 | *Commensalibacter melissae* | CP046393.1 | 97.25% |
|  | *Exoneurella tridentata* | SMST01003845 | *Commensalibacter intestini* | LT631761.1 | 96.66% |
| *Pseudomonas* sp. | *Exoneurella tridentata* | SMST01005604 | *Pseudomonas koreensis* | CP155621.1 | 98.35% |
| *Sodalis* sp. | *Exoneurella tridentata* | SMST01007755 | *Sodalis praecaptivus* | CP006569.1 | 98.51% |
|  |  |  |  |  |  |
| **Microsporidian parasite** |  |  |  |  |  |
| unknown microsporidium | *Exoneura robusta* | SMSS01004782 | *Alteronosema astaquatica* | OM501730 | 95.48% |
|  |  |  |  |  |  |
| **Protozoan parasite** |  |  |  |  |  |
| unknown gregarine | *Exoneurella tridentata* | SMST01005406 | *Gregarina niphandrodes* | FJ459747.1 | 77.61% |
|  |  |  |  |  |  |

**Table S3. Cobiont screens and matches.** Existing genome projects for *Exonera robusta* (GCA_019453415.1) and *Exoneurella tridentata* (GCA_019453975.1) were screened for small subunit ribosomal ribonucleic acid (SSU rRNA) genes of target microbes. Identified contigs were used to search (BlastN) for matches in GenBank for which the best hit and sequence match (%) between allodapine microbe contig and GenBank-hit are listed.

**Table S4. Angiosperms visited by allodapine bees in natural landscapes.**

| PLANT |  |  | BEE |  |  |  |  |  |  |  |
| --- | --- | --- | --- | --- | --- | --- | --- | --- | --- | --- |
| Family | Genus | Cont | *Allodape* | *Allodapu* | *Brauns.* | *Comp.* | *Exon.* | Brev. | *Exlla*. | Ref |
| Aizoaceae | *Bergeranthus* | AF |  | ⚫ |  |  |  |  |  | 5 |
| Amaranthaceae | *Aerva* | AU |  |  | ⚫ |  |  |  |  | 17 |
|  | *Ptilotus* | AU |  |  | ⚫ |  |  |  |  | 17 |
| Apiaceae | *Trachymene* | AU |  |  |  |  | ⚫ |  |  | 17 |
| Asteraceae | *Helipterum* | AU |  |  |  |  | ⚫ |  |  | 17 |
| Boraginaceae | *Trichodesma* | AU |  |  | ⚫ |  |  |  |  | 17 |
| Caesalpiniaceae | *Senna* | AU |  |  | ⚫ |  |  |  |  | 17 |
| Chenopodiaceae | *Rhagodia* | AU |  |  |  |  | ⚫ |  |  | 17 |
| Chloranthaceae | *Dicrastylis* | AU |  |  |  |  | ⚫ |  |  | 17 |
| Iridaceae | *Gladiolus* | AF | ⚫ |  |  |  |  |  |  | 1,2 |
| Epacridaceae | *Leucopogon* | AU |  |  |  |  | ⚫ |  |  | 17 |
| Ericaceae | *Woollsia* | AU |  |  | ⚫ |  |  |  |  | 11 |
| Fabaceae | *Humboldtia* | AS |  |  | ⚫ |  |  |  |  | 9,10 |
|  | *Dillwynia* | AU |  |  | ⚫ |  |  |  |  | 11 |
| Goodeniaceae | *Scaevola* | AU |  |  |  |  | ⚫ |  | ⚫ | 17 |
| Lamiaceae | *Prostanthera* | AU |  |  |  |  |  |  | ⚫ | 18 |
| Malvaceae | *Malvastrum* | AU |  |  |  |  |  |  | ⚫ | 17 |
| Marantaceae | *Marantochloa* | AF |  | ⚫ |  |  |  |  |  | 6 |
|  | *Sarcophrynium* | AF |  | ⚫ |  |  |  |  |  | 6 |
| Mimosaceae | *Acacia* | AU |  |  | ⚫ |  |  |  |  | 17 |
| Myoporaceae | *Eremophila* | AU |  |  | ⚫ |  | ⚫ | ⚫ | ⚫ | 17 |
| Myrtaceae | *Agonis* | AU |  |  |  |  | ⚫ |  |  | 17 |
|  | *Corymbia* | AU |  |  |  |  | ⚫ |  |  | 17 |
|  | *Eremaea* | AU |  |  |  |  | ⚫ |  |  | 17 |
|  | *Eucalyptus* | AU |  |  | ⚫ |  | ⚫ | ⚫ | ⚫ | 17 |
|  | *Leptospermum* | AU |  |  |  |  | ⚫ |  |  | 17 |
|  | *Melaleuca* | AU |  |  | ⚫ |  | ⚫ | ⚫ |  | 17 |
|  | *Thryptomene* | AU |  |  |  |  | ⚫ |  |  | 17 |
|  | *Verticordia* | AU |  |  |  |  | ⚫ |  |  | 17 |
| Orchidaceae | *Diuris* | AU |  |  |  |  | ⚫ |  |  | 16 |
|  | *Thelymitra* | AU |  |  |  |  | ⚫ |  |  | 14 |
|  | *Vanilla* | AF | ⚫ | ⚫ |  |  |  |  |  | 3,4 |
| Papilonaceae | *Bossiaea* | AU |  |  |  |  | ⚫ |  |  | 17 |
|  | *Crotalaria* | AU |  |  |  |  |  |  | ⚫ | 17 |
|  | *Daviesia* | AU |  |  |  |  |  |  | ⚫ | 17 |
|  | *Jacksonia* | AU |  |  |  |  | ⚫ |  |  | 17 |
|  | *Gastrolobium* | AU |  |  |  |  | ⚫ |  |  | 17 |
| Poaceae | *Melocanna* | AS |  |  | ⚫ |  |  |  |  | 12 |
|  | *Ochlandra* | AS |  |  | ⚫ |  |  |  |  | 8 |
| Portulacaceae | *Calandrinia* | AU |  |  | ⚫ |  |  |  |  | 17 |
| Proteaceae | *Dryandra* | AU |  |  | ⚫ |  | ⚫ |  | ⚫ | 17 |
|  | *Grevillea* | AU |  |  | ⚫ |  | ⚫ |  | ⚫ | 17 |
|  | *Persoonia* | AU |  |  |  |  | ⚫ |  |  | 17 |
| Rutaceae | *Boronia* | AU |  |  | ⚫ |  |  |  |  | 11 |
|  | *Philotheca* | AU |  |  | ⚫ |  |  |  |  | 11 |
| Sapotaceae | *Vitellaria* | AF |  |  |  | ⚫ |  |  |  | 13 |
| Solanaceae | *Solanum* | AU |  |  | ⚫ |  | ⚫ |  |  | 7,17 |
| Tiliaceae | *Corchorus* | AU |  |  | ⚫ |  |  |  |  | 17 |
| Trimeniaceae | *Trimenia* | AU |  |  |  |  | ⚫ |  |  | 15 |
| Xanthorrhoeaceae | *Xanthorrhoea* | AU |  |  |  |  | ⚫ |  |  | 17 |

**Table S4. Angiosperms visited by allodapine bees in natural landscapes.** Continent abbreviations: Africa (AF); Asia (AS); Australia (AU). Abbreviated column headings for Continent (Cont.) and allodapine bee genera: *Allodapula* (*Allodapu*.); *Braunsapis* (*Brauns*.); *Compsomelissa* (*Compso*.); *Exoneura* (*Exon*.); *Brevineura* (*Brev*.); *Exoneurella* (*Exlla*.) . Cited references: 1. Goldblatt et al. 1997.; 2. Goldblatt et al. 1998; 3. Gigant et al. 2014; 4. Gigant et al. 2016; 5. Peter et al. 2004; 6. Ley & Claßen-Bockhoff 2009; 7. Anderson & Symon 1988; 8. Koshy et al. 2001; 9. Shenoy & Borges 2008; 10. Dev et al. 2010; 11. Elliott et al. 2021; 12. Koshy et al. 2022; 13. Lassen et al. 2018; 14. Sydes & Calder 1993; 15. Bernhardt et al. 2003; 16. Indsto et al. 2007; 17. Houston 2000; 18. Bernhardt & Weston 1996.

**References**

Aidoo, K.S. 2008. Boosting cashew production in Ghana. *Bees for Development Journal* 91: 8-9.

Alberoni, D., Gaggìa, F., Baffoni, L., Modesto, M.M., Biavati, B., Di Gioia, D. 2019. *Bifidobacterium xylocopae* sp. nov. and *Bifidobacterium aemilianum* sp. nov., from the carpenter bee (*Xylocopa violacea*) digestive tract. *Systematic and Applied Microbiology* 42: 205-216.

Anderson, G.J., Symon, D. 1988. Insect foragers on *Solanum* flowers in Australia. *Annals of the Missouri Botanical Garden* 75: 842-852.

Batra, S.W.T. 1967. Crop pollination and the flower relationships of the wild bees of Ludhiana, India (Hymenoptera: Apoidea). *Journal of the Kansas Entomological Society* 40: 164-177.

Batra, S.W.T. 1976. Nests of *Ceratina*, *Pithitis* and *Braunsapis* from India (Hymenoptera: Anthophoridae). *Oriental Insects* 10: 1-9.

Batra, S.W.T. 1977. Bees of India (Apoidea), their behaviour, management and a key to the genera. *Oriental Insects* 11: 289-324.

Batra, S.W.T. 1997. Fruit pollinating bees of the Garhwal Himalaya, U.P., India. *Acta Horticulturae* 437: 325-328.

Bernauer OM, Branstetter MG, Cook JM & Tierney SM. 2024. Functional trait mismatch between native and introduced bee pollinators servicing a global fruit crop. *BMC Ecology & Evolution* 24: 104

Bernauer, O.M., Cook, J.M., Tierney, S.M. 2021. Nesting biology and social organisation of the allodapine bee *Exoneura angophorae* (Hymenoptera: Apidae): montane environmental constraints yield biased sex allocation yet phenology is unhindered. *Insectes Sociaux* 68: 337-349.

Bernauer, O.M., Cook, J.M., Tierney, S.M. 2022a. Division of foraging behaviour: assessments of pollinator traits when visiting a model plant species. *Animal Behaviour* 188: 169-179.

Bernauer, O.M., Tierney, S.M., and Cook, J.M. 2022b. Efficiency and effectiveness of native bees and honey bees as pollinators of apples in New South Wales orchards. *Agriculture, Ecosystems & Environment* 337: 108063.

Bernhardt, P., Sage, T., Weston, P., Azuma, H., Lam M., Thien, L.B., Bruhl, J. 2003. The pollination of *Trimenia moorei* (Trimeniaceae): floral volatiles, insects/wind pollen vectors and stigmatic self-incompatibility in a basal angiosperm. *Annals of Botany* 92: 445-458.

Bernhardt, P., and Weston, P.H. 1996. The pollination ecology of *Persoonia* (Proteaceae) in eastern Australia. *Telopea* 6: 775-804.

Brettell, L.E., Riegler, M., O'Brien, C., Cook, J.M., 2020. Occurrence of honey bee-associated pathogens in *Varroa*-free pollinator communities. *Journal of Invertebrate Pathology* 171: 107344.

Brown, J., Barton, P.S. Cunningham, S.A. 2020. Flower visitation and land cover associations of above ground- and below ground-nesting native bees in an agricultural region of south-east Australia. *Agriculture, Ecosystems & Environment* 295: 106895.

Brown, J., Groom, S.V.C., Rader, R., Hogendoorn, K., Cunningham, S.A. 2022. Land cover associations of wild bees visiting flowers in apple orchards across three geographic regions of southeast Australia. *Agriculture Ecosystem & Environment* 324: 107717.

Chau, K.D., Samad-zada, F., Kelemen, E.P., Rehan, S.M. 2023. Integrative population genetics and metagenomics reveals urbanization increases pathogen loads and decreases connectivity in a wild bee. *Global Change Biology* 29: 4193-4211.

Coates, J.M., Brown, J., and Cunningham, S.A. 2022. Wild bees nest in the stems of cultivated *Rubus* plants and act as effective crop pollinators. Agriculture, Ecosystems & Environment 325: 107741.

Dev, S.A., Shenoy, M., Borges, R.M. 2010. Genetic and clonal diversity of the endemic ant-plant *Humboldtia brunonis* (Fabaceae) in the Western Ghats of India. *Journal of Bioscience* 35: 267-279.

Dew, R.M., McFrederick, Q.S., Rehan, S.M. 2020. Diverse diets with consistent core microbiome in wild bee pollen provisions. *Insects* 11: 499.

Elliott, B., Wilson, R., Shapcott, A., Keller, A., Newis, R., Cannizzaro, C., Burwell, C., Smith, T., Leonhardt, S.D., Kämper, W. Wallace, H.W. 2021. Pollen diets and niche overlap of honey bees and native bees in protected areas. *Basic and Applied Ecology* 50: 169-180.

Fernandez De Landa, G., Alberoni, D., Baffoni, L., Fernandez De Landa, M., Revainera, P.D., Porrini, L.P., Brasesco, C., Quintana, S., Zumpano, F., Eguaras, M.J., Matias Daniel Maggi, M.D., Di Gioia, D. 2023. The gut microbiome of solitary bees is mainly affected by pathogen assemblage and partially by land use. *Environmental Microbiome* 18: 38.

Gigant, R.L., de Bruyn, A., Church, B., Humeau, L., Gauvin-Bialecki, A., Pailler, T., Grisoni M.,Besse, P. 2014. Active sexual reproduction but no sign of genetic diversity in range-edge populations of *Vanilla* *roscheri* Rchb. f. (Orchidaceae) in South Africa. *Conservation Genetics* 15: 1403-1415.

Gigant, R.L., de Bruyn, A., M'sa, T., Viscardi, G., Gigord, L., Gauvin-Bialecki, A., Pailler, T., Humeau, L., Grisoni, M., Besse, P. 2016. Combining pollination ecology and fine-scale spatial genetic structure analysis to unravel the reproductive strategy of an insular threatened orchid. *South African Journal of Botany* 105: 25-35.

Gilliam, M., Lorenz B.J., Buchmann, S.L. 1994. *Ascosphaera apis*, the chalkbrood pathogen of the honey bee, *Apis mellifera*, from larvae of a carpenter bee *Xylocopa californica arizonensis*. *Journal of Invertebrate Pathology* 63: 307-309.

Goldblatt, P., Manning, J.C., Bernhardt, P. 1997. Notes on the pollination of *Gladiolus brevifolius* (Iridaceae) by bees (Anthophoridae) and bee mimicking flies (Psilodera: Acroceridae). *Journal of the Kansas Entomological Society* 70: 297-304.

Goldblatt, P., Manning, J.C., Bernhardt, P. 1998. Adaptive radiation of bee-pollinated *Gladiolus* species (Iridaceae) in Southern Africa. *Annals of the Missouri Botanical Garden* 85: 492-517.

Graystock, P., Rehan, S.M., McFrederick, Q.S. 2017. Hunting for healthy microbiomes: determining the core microbiomes of *Ceratina*, *Megalopta*, and *Apis* bees and how they associate with microbes in bee collected pollen. *Conservation Genetics* 18: 701-711.

Gu, Y., Han, W., Wang, Y., Liang, D., Gao, J., Zhong, Y., Zhao, S., Wang, S. 2023. *Xylocopa caerulea* and *Xylocopa auripennis* harbor a homologous gut microbiome related to that of eusocial bees. *Frontiers in Microbiology* 14: 1124964.

Handy, M.Y., Sbardellati, D.L., Yu, M., Saleh, N.W., Ostwald, M.M., Vannette, R.L. 2023. Incipiently social carpenter bees (*Xylocopa*) host distinctive gut bacterial communities and display geographical structure as revealed by full-length PacBio 16S rRNA sequencing. *Molecular Ecology* 32: 1530-1543.

Holley, J.A.C., Jackson, M.N., Pham, A.T., Hatcher, S.C., Moran, N.A. 2022. Carpenter bees (*Xylocopa*) harbor a distinctive gut microbiome related to that of honey bees and bumble bees. *Applied and Environmental Microbiology* 88: e00203-22.

Houston, TF. 2000. *Native bees on wildflowers in Western Australia*. Western Australian Insect Study Society: Perth.

Indsto, J.O., Weston, P.H., Clements, M.A., Dyer, A.G., Batley, M., Whelan, R.J. 2007. Generalised pollination of *Diuris alba* (Orchidaceae) by small bees and wasps. *Australian Journal of Botany* 55: 628-634.

Jaboor, S.K., da Silva, C.R.B., Kellermann, V. 2022. The effect of environmental temperature on bee activity at strawberry farms. *Austral Ecology* 47: 1470-1479.

Kaliaperumal, V., Gupta, A., Thiruvengadam, V., Pannure, A., Raghavendra, A.T. 2022. Biological notes on nesting biology, development and natural enemies of *Braunsapis mixta*, a pollinator of cashew. *Journal of Apicultural Research* 62: 1132-1144.

Koshy, K.C., Harikumar, D., Narendran T.C. 2001. Insect visits to some bamboos of the Western Ghats, India. *Current Science* 81: 833-838.

Koshy, K.C. Gopakumar, B., Sebastian, A.S., Nair S., A., Johnson, A.J., Govindan, B., Baby, S. 2022. Flower fruit dynamics, visitor-predator patterns and chemical preferences in the tropical bamboo, *Melocanna baccifera*. *PLoS ONE* 17: e0277341.

Lassen, K.M., Nielsen, L.R., Lompo, D. Dupont, Y.L., Kjær, E.D. 2018. Honey bees are essential for pollination of *Vitellaria paradoxa* subsp. *paradoxa* (Sapotaceae) in Burkina Faso. *Agroforestry Systems* 92: 23-34.

Ley, A.C., Claßen-Bockhoff, R. 2009. Pollination syndromes in African Marantaceae. *Annals of Botany* 104: 41-56.

Lucia, M., Reynaldi, F.J., Sguazza, G.H., Abrahamovich, A.H. 2014. First detection of deformed wing virus in *Xylocopa augusti* larvae (Hymenoptera: Apidae) in Argentina. *Journal of Apicultural Research* 53: 466-468.

McFrederick, Q.S., Rehan, S.M. 2016. Characterization of pollen and bacterial community composition in brood provisions of a small carpenter bee. *Molecular Ecology* 25: 2302-2311.

Mee, L., Barribeau, S.M. 2023. Influence of social lifestyles on host-microbe symbioses in the bees. *Ecology and Evolution* 13: e10679.

Nguyen, P.N., Rehan, S.M. 2022a. Developmental microbiome of the small carpenter bee, *Ceratina calcarata*. *Environmental DNA* 4: 808-819.

Nguyen, P.N., Rehan, S.M. 2022b. The effects of urban land use gradients on wild bee microbiomes. *Frontiers in Microbiology* 13: 992660.

Nguyen, P.N., Rehan, S.M. 2023. Wild bee and pollen microbiomes across an urban–rural divide. *FEMS Microbiology Ecology* 99: fiad158.

Peter, C.I., Dold, A.P., Barker, N.P., Ripley, B.S. 2004. Pollination biology of *Bergeranthus multiceps* (Aizoaceae) with preliminary observations of repeated flower opening and closure. *South African Journal of Science* 100: 624-629.

Plischuk, S., Quintana, S., Fernandez de Landa, G., Revainera, P.D., Haramboure, M., Lange, C.E. 2023. Detection of *Apicystis bombi* (Apicomplexa: Neogregarinorida) in carpenter bees of Argentina. *International Journal for Parasitology: Parasites and Wildlife* 21: 43-46.

Prendergast, K.S., Leclercq, N., Vereecken, N.J. 2021. Honey bees (Hymenoptera: Apidae) outnumber native bees in Tasmanian apple orchards: perspectives for balancing crop production and native bee conservation. *Austral Entomology* 60: 422-435.

Quintana, S., Fernandez de Landa, G., Revainera, P., Meroi, F., Porrini, L., Di Geronimo, V., Brasesco, C., Plischuk, S., Eguaras, M.J., Maggi, M. 2019. Broad geographic and host distribution of *Apis mellifera* filamentous virus in South American native bees. *Journal of Apicultural Science* 63: 327-332.

Radzevičiūtė, R., Theodorou, P., Husemann, M., Japoshvili, G., Kirkitadze, G., Zhusupbaeva, A., Paxton, R.J. 2017. Replication of honey bee-associated RNA viruses across multiple bee species in apple orchards of Georgia, Germany and Kyrgyzstan. *Journal of Invertebrate Pathology* 146: 14-23.

Reynaldi, F.J, Lucia, M., Genchi Garcia, M.L. 2015. *Ascosphaera apis*, the entomopathogenic fungus affecting larvae of native bees (*Xylocopa augusti*): first report in South America. *Revista Iberoamericana de Micología* 32: 261-264.

Santamaria, J. Villalobos, E.M., Brettell, L.E., Nikaido, S., Graham, J.R., Martin, S. 2018. Evidence of *Varroa*-mediated deformed wing virus spillover in Hawaii. *Journal of Invertebrate Pathology* 151: 126-130.

Shenoy, M., Borges, R.M. 2008. A novel mutualism between an ant-plant and its resident pollinator. *Naturwissenschaften* 95: 61–65.

Singh, R., Levitt, A.L., Rajotte, E.G., Holmes, E.C., Ostiguy, N., van Engelsdorp, D., Lipkin, W.I., de Pamphilis, C.W., Toth, A.L., Cox-Foster, D.L. .2010. RNA viruses in hymenopteran pollinators: evidence of inter-taxa virus transmission via pollen and potential impact on non-*Apis* hymenopteran species. *PLoS ONE* 5: e14357.

Singh, G., Makinson, J.C., Gilpin, A.M., Spooner-Hart, R.N., Cook, J.M. 2024. Wild native insects are efficient pollinators of mangoes in the Northern Territory of Australia. *Agriculture Ecosystems & Environment* 374: 109161.

Stow, A., Turnbull, C., Gillings, M., Smith, S., Holley, M., Silberbauer, L., Wilson, P.D., Briscoe, D., and Beattie, A. 2010. Differential antimicrobial activity in response to the entomopathogenic fungus Cordyceps in six Australian bee species. *Australian Journal of Entomology* 49: 145-149.

Subta, P., Yodsuwan, P., Yongsawas, R., In-on, A., Warrit, N., Panha, S., Khongphinitbunjong, K., Chantawannakul, P., Attasopa, K., Disayathanoowat, T. 2020. Bacterial communities in three parts of intestinal tracts of carpenter bees (*Xylocopa tenuiscapa*). *Insects* 11: 497.

Sydes, M.A., Calder, D.M. 1993. Comparative reproductive biology of two sun-orchids; the vulnerable *Thelymitra circumsepta* and the widespread *T. ixioides* (Orchidaceae). *Australian Journal of Botany* 41: 577-89.

Tierney, S.M., Bernauer, O.M., King, L., Spooner-Hart, R., Cook, J.M. 2023. Bee pollination services and the burden of biogeography. *Proceedings of the Royal Society of London B* 290: 20230747.

Tierney, S.M., Jeffries, T.C., Koch, H. 2025. Data from: Microbial evolution in allodapine bees: perspectives from trophallactic, socially plastic pollinators. *Xxxxxxxx University research data store*. (doi: in preparation)

Vanitha, K., Raviprasad, T.N. 2019. Diversity, species richness and foraging behaviour of pollinators in cashew. *Agricultural Research* 8: 197-206.
